# Safety of Prolonged Exposure of Human Skin to Aqueous Ozone in a Test Hand Hygiene Model

**DOI:** 10.1111/srt.13675

**Published:** 2024-04-01

**Authors:** Brian R. Leon, Robert M. Lubitz, Sarah A. Landsberger, Hannah D. Buckingham, Rachael A. Hiday, K. Nicole Bradner, Catherine H. Adaniya

**Affiliations:** ^1^ School of Medicine Indiana University Indianapolis Indiana USA; ^2^ John Sealy School of Medicine University of Texas Medical Branch Galveston Texas USA; ^3^ Academic Health Center Pharmacy Indiana University Health Indianapolis Indiana USA

**Keywords:** Aqueous ozone, hand hygiene, infection control, repeated exposures

## Abstract

**Aims:**

This research assessed the safety of aqueous ozone (AO) on human skin after multiple exposures for up to 40 hours.

**Methods and Results:**

Full thickness recombinant human skin (EpiDerm FT, EFT‐400) was exposed to AO for 7 seconds per minute for the first 6 minutes of each hour, repeated hourly over four time periods (4, 10, 20 and 40 hours). An MTT assay assessed viability of skin cells after exposure, compared to incubator control, negative control and vehicle control (distilled water). No significant difference in tissue viability was found between the AO condition and any of the control conditions through 20 hours of exposures. At 40 hours of exposure, tissue viability was lower in the AO group when compared with negative control (p = 0.030) but not the other controls.

**Conclusions:**

The current study supports further consideration of repeated application of AO on human skin, such as for hand hygiene.

**Impact Statement:**

The present research is the first well‐controlled in vitro study assessing the cytotoxicity of repeated exposures of AO on a full‐thickness human skin model. This information helps to inform the evaluation of AO as a potential alternative for hand and wound antisepsis.

## 1 INTRODUCTION

Aqueous ozone (AO) has gained attention as a potential safe and effective alternative for hand antisepsis.^1^ Ozone (O_3_) is an inorganic and highly reactive gas composed of three oxygen atoms. AO is the dissolved form produced by one of two methods: (1) coronal discharge, where an electrical charge is applied to pure oxygen gas or air to create O_3_ gas, which is then incorporated with water, or (2) direct water electrolysis using low voltage applied to water flowing across a polymer membrane in a compact electrolytic cell.^2^ The AO rapidly decomposes upon contact with naturally occurring organic materials, returning to free oxygen and water while releasing the free radicals responsible for disinfection.^3^, ^4^

The disinfection properties of ozone have been known since its discovery in the mid‐1800s,^5^ and AO has been used for disinfection across multiple industries, including food, water treatment, pharmaceuticals, textiles, healthcare, and the medical sector.^6^, ^7^, ^8^, ^9^ AO has shown great promise as an effective and renewable alternative for hand hygiene.

The cytotoxic effect of repeated and prolonged AO exposure to human skin, such as what would occur with clinical hand hygiene protocols, is unclear. In vitro cytotoxicity studies of short exposure of AO in a human keratinocytes model resulted in a cell survival rate of 92.4% with no damage to, or below, the stratum corneum.^10^ However, we have been unable to identify any cellular viability studies using full‐thickness skin models following prolonged, repeated exposures typical of healthcare hand hygiene practices.^11^ This trial evaluated the cytotoxicity of AO on human skin while simulating the multiple exposures utilized for hand hygiene as in a healthcare setting using a novel hand‐hygiene device modified for this study.^12^

## 2 MATERIALS AND METHODS

**FIGURE 1:**
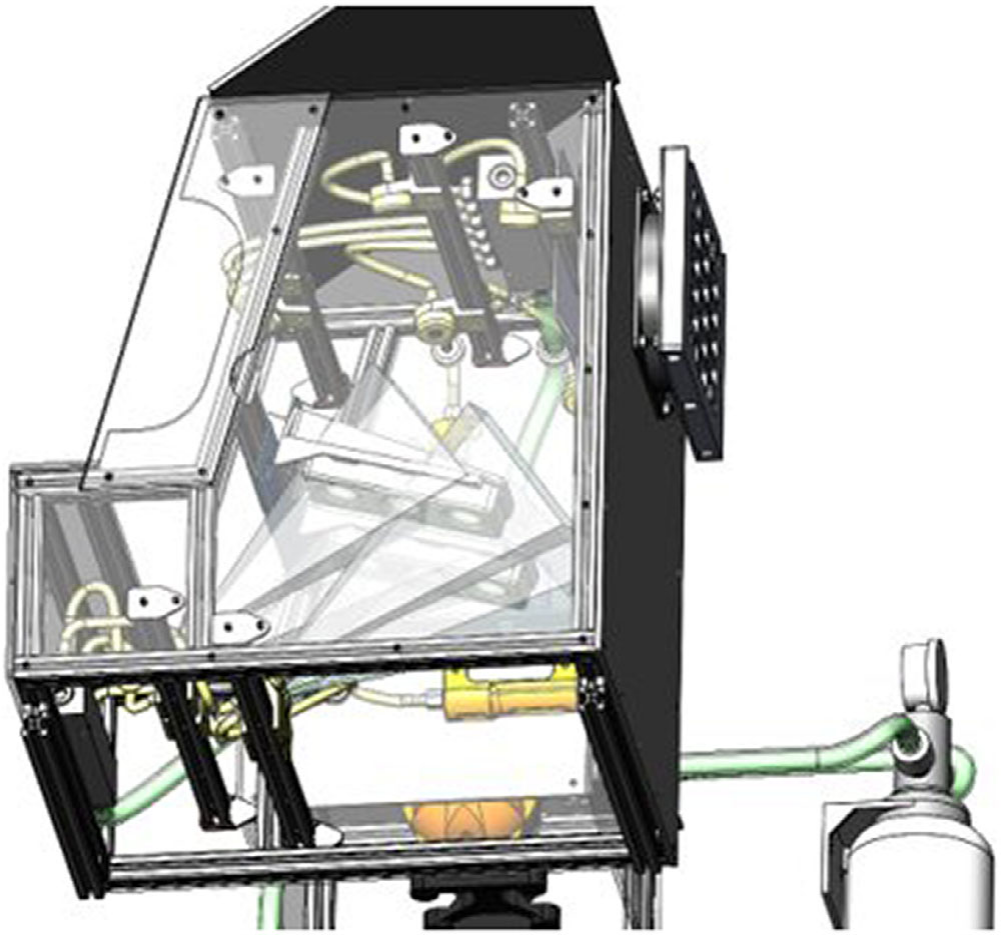
V0 Spray Booth Assembly. Note: Image provided by Mat Tek Corporation (Massachusetts, USA).

This study used a recombinant human skin model (EpiDermFT^TM^ EFT‐400, MatTek Corporation, Ashland, MA, USA), consisting of in vivo‐like epidermis and dermis produced from normal human‐derived epidermal keratinocytes and normal human‐derived dermal fibroblasts. This model is commonly used as a means to test for dermal irritation and cytotoxicity. Tissue samples (“ETF‐400”) were exposed to AO using a novel hand hygiene device described in more detail elsewhere^12^ with a custom‐made tissue holder (“holder”). The device created a consistent concentration of 4 ppm ozone in water, gently sprayed on the tissue specimens using uniform 1mm droplets for a 7 second cycle. The hand hygiene device was modified (“Spray Chamber”) to ensure proper position of the holder so that tissue would be directly exposed to the AO spray (See Figure 1).

Exposure frequencies were selected based on Maximal Usage Trial (MUsT) guidance provided by the U.S. Food and Drug Administration and the projected maximum number of handwashes by healthcare workers in a specified interval of time.^13^ Specifically, test times per group were 4 hours, 10 hours, 20 hours, and 40 hours (see Table 1). Six exposures occurred each hour, resulting in 24, 60, 120, or 240 exposures per group. Exposures occurred in the first six minutes of each hour for the first 7 seconds of each minute.

**TABLE 1:** Group conditions, test periods and number of exposures.

Group #	Exposure Condition	Test Period	Total # of Exposures (7 sec. duration per exposure)	Total Exposure Time	# of Tissues
1	Incubator Control (IC 1)	4 hours	–	–	3
2	Incubator Control (IC 2)	10 hours	–	–	3
3	Incubator Control (IC 3)	20 hours	–	–	3
4	Incubator Control (IC 4)	40 hours	–	–	3
5	Negative Control (NC 1)	4 hours	24	168 sec.	3
6	Negative Control (NC 2)	10 hours	60	420 sec.	3
7	Negative Control (NC 3)	20 hours	120	840 sec	3
8	Negative Control (NC 4)	40 hours	240	1680 sec.	3
9	Vehicle Control (VC 1)	4 hours	24	168 sec.	3
10	Vehicle Control (VC 2)	10 hours	60	420 sec.	3
11	Vehicle Control (VC 3)	20 hours	120	840 sec.	3
12	Vehicle Control (VC 4)	40 hours	240	1680 sec.	3
13	Aqueous Ozone (AO 1)	4 hours	20	168 sec.	3
14	Aqueous Ozone (AO 2)	10 hours	60	420 sec.	3
15	Aqueous Ozone (AO 3)	20 hours	120	840 sec	3
16	Aqueous Ozone (AO 4)	40 hours	240	1680 sec.	3

*Notes*:

Incubator Control (IC)—tissues remained in the incubator for the entire test period.

Negative Control (NC)—tissues removed from incubator but were untreated.

Vehicle Control (VC)—tissues removed from incubator and exposed to dH_2_0.

Aqueous Ozone (AO)—tissues removed from incubator and exposed to AO.

Test period of 20‐hours was divided into 2, 10‐hour test periods on 2 consecutive days

Test period of 40‐hours was divided into 4, 10‐hour test periods on 4 consecutive days.

**FIGURE 2:**
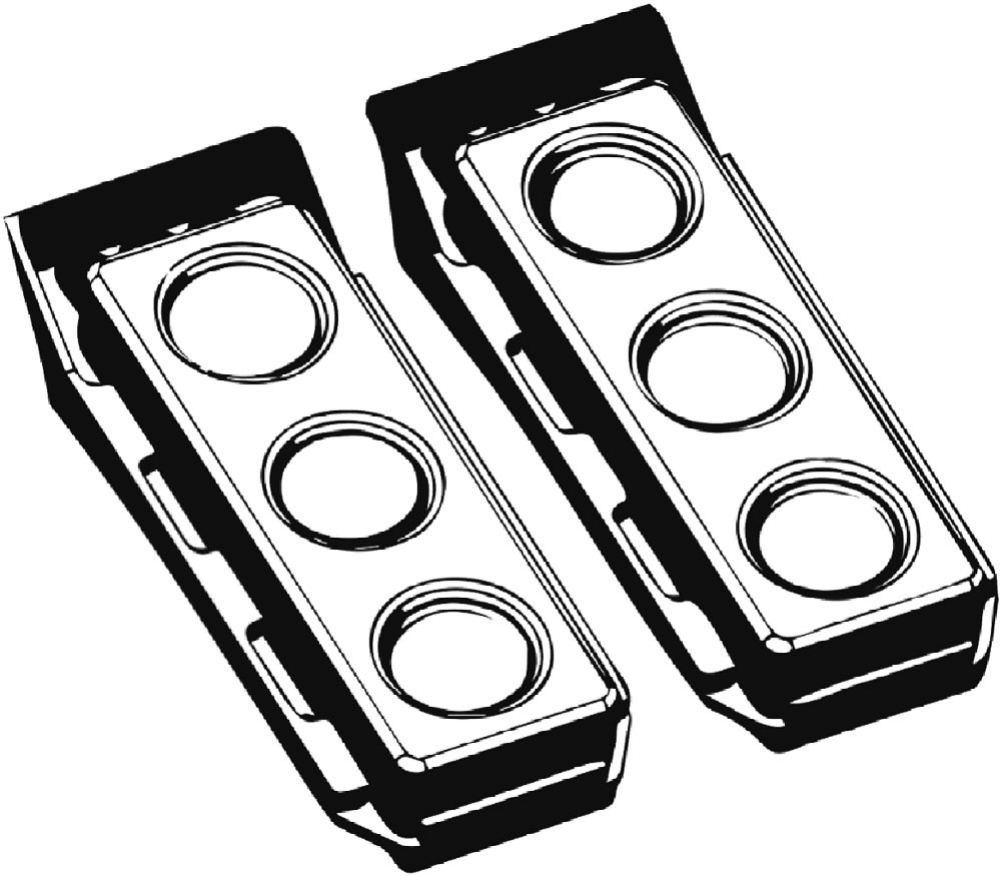
Well plates for tissue samples. Note: Graphic created by Brian Leon.

All tissues were incubated at 37°C/5% CO_2_ prior to and between each test. The tissues for each of the 16 groups were prepared on a unique 6‐well plate (see Figure 2). The wells were filled with 5mL of culture media, and the EFT‐400 tissue was transferred to the prepared wells. Cell culture media was changed every other day. Consistent with standard procedures, penicillin/streptomycin was administered to the three wells for each group tested for 40 hours following the final test period at a concentration of 1% (%v/v) to prevent bacterial growth that could confound MTT test results.

As seen in Table 1, we created a test matrix of 16 groups to allow test and control samples. Each group had three ETF‐400 samples. Incubator controls (Groups 1–4) were not removed from the incubator but were collected at their designated 4‐hour, 10‐hour, 20‐hour, and 40‐hour times alongside tissues from groups 5–16 with the same length of exposure. Following the hourly exposure schedule, groups 5–16 were removed from the incubator immediately before scheduled exposures.

Negative controls (Groups 5–8) were removed from the incubator using sterile methods. The EFT‐400 tissue was placed into a unique carrier designed to hold the plates in the Spray Chamber. Instead of placement into the Spray Chamber, the carrier was placed in a biological safety cabinet for the six 7‐second sessions per hour for 4 hours, 10 hours, 20 hours, or 40 hours as a “mock exposure.”

Exposed tissue in groups 9–16 were considered the treated condition, either with distilled water (dH_2_0) or AO. These specimens transferred to a unique carrier designed to hold the plates in the Spray Chamber using sterile methods. A cover was placed on the carrier for transport into the Spray Chamber assembly and then removed. Exposure commenced once the Spray Chamber hood was closed. Tissues remained in the Spray Chamber until the six exposures were complete. After removing the tissue from the Spray Chamber, the spray system was purged three times using d H_2_0 prior to the next test.

Vehicle controls (Groups 9–12) were exposed to six 7‐second sprays of dH_2_0 per hour for 4 hours, 10 hours, 20 hours, or 40 hours. Tissues exposed to AO (Groups 13–16) were exposed to six 7‐second sprays of AO (4.0 ppm) per hour for 4 hours, 10 hours, 20 hours, or 40 hours.

Following exposures for groups 9–16, the carrier was removed from the Spray Chamber. A cover was reattached for transport and then removed after the carrier was placed in a biological safety cabinet. The tissue surfaces were aspirated in the safety cabinet for groups 5–16 to remove residual test material. Tissue bottom surfaces were blotted with sterile gauze before being returned to culture media. The 6‐well plates stayed in the incubator overnight (14‐18 hours) following the final exposure of the test period. The Spray Chamber assembly water tank was emptied and refilled each day with d H_2_0.

Following exposure, viability of ozone‐exposed tissues was assessed using an MTT cytotoxicity assay.^14^ Tissues were placed into MTT reagent‐containing wells and incubated at 37°C with 5% CO_2_ for 3 hours. Following incubation, tissues were removed from the MTT and blotted dry. Sterile forceps were used to separate the dermis and epidermis manually. Each layer was placed into a separate well in a sterile 6‐well plate, and 4mL of extractant solution was added. Extraction was conducted overnight at room temperature or for 2 hours at room temperature on a shaker. Plates were sealed to prevent extractant evaporation and light exposure. Following extraction, tissues were removed and discarded.

The remaining extractant solution was mixed thoroughly, and then 200 µL from each sample was added to a 96‐well plate. The optical density (OD) of each sample was measured at 570 nm (650 nm wavelength correction). For the blank, 200 µL of extractant solution was used. Whole tissue viability was measured as a sum of the individual optical densities of the epidermis and dermis. Tissue viability calculation was determined relative to the Incubator Control (IC), Negative Control (NC), and Vehicle Control (VC; dH_2_O) according to the following formulas: % viability (relative to IC) = [OD/ AVG ODIC] x 100; % viability (relative to NC) = [OD/ AVG ODNC] x 100; % viability (relative to VC) = [OD/ AVG ODVC] x 100. Student's t‐tests were used to compare treatment conditions of every exposure length (24 exposures, 60 exposures, 120 exposures, 240 exposures) to each control (IC, NC, VC) to determine significant differences between groups. A p‐value of 0.05 or less was used as the cut‐off for statistical significance.

## 3 RESULTS

The raw optical density (OD) mean and standard deviation for each treatment condition and duration are presented in Table 2. Cellular viability was calculated relative to each control condition (Tables 3, 4, 5). Compared to IC (Table 3), the AO‐exposed tissues had similar viability over all measurement periods. This supports the incubator process did not confound viability. Compared to NC (Table 4), AO‐exposed tissues had 85% viability after 40 hours, which was lower than NC (p = 0.030). Finally, AO‐exposed tissues had similar viability to VC tissues throughout the study (Table 5). These results suggest no, or at most, limited cytotoxic effect of AO following prolonged exposure.

**TABLE 2:** Raw optical density values by condition and number of exposures.

Treatment Condition	24 exposures over 4hrs		60 exposures over 10 hours		120 exposures over 20 hours		240 exposures over 40 hours	
	Mean	SD	Mean	SD	Mean	SD	Mean	SD
**IC**	2.139	0.060	2.180	0.074	2.329	0.046	2.086	0.045
**NC**	2.110	0.035	2.301	0.133	2.412	0.088	2.326	0.095
**VC**	2.130	0.068	2.099	0.044	2.562	0.144	1.803	0.034
**AO**	2.127	0.104	2.049	0.051	2.449	0.090	1.984	0.146

*Note*: n = 3 tissues per treatment condition and number of exposures

**TABLE 3:** Percent viability by treatment condition and number of exposures vs. incubator control (IC)

Treatment Condition	24 exposures over 4hrs		60 exposures over 10 hours		120 exposures over 20 hours		240 exposures over 40 hours	
	Mean %	SD	Mean %	SD	Mean %	SD	Mean %	SD
**IC**	100.000	2.811	100.00	3.413	100.00	1.981	100.000	2.140
**NC**	98.660	1.628	105.550	6.107	103.564	3.778	111.505^*^	4.534
**VC**	99.610	3.179	96.285	2.010	110.019	6.172	86.433^*^	1.640
**AO**	99.470	4.871	93.992	2.326	105.152	3.865	95.126	6.987

*Notes*: n = 3 tissues per treatment condition and number of exposures;

* = p‐value <0.05

**TABLE 4:** Percent viability by treatment condition and number of exposures vs. negative control (NC)

Treatment Condition	24 exposures over 4hrs		60 exposures over 10 hours		120 exposures over 20 hours		240 exposures over 40 hours	
	Mean %	SD	Mean %	SD	Mean %	SD	Mean %	SD
**IC**	101.359	2.849	94.742	3.233	96.559	1.913	89.682^*^	1.919
**NC**	100.000	1.651	100.000	5.786	100.000	3.648	100.000	4.066
**VC**	100.964	3.222	91.222	1.904	106.233	5.959	77.515^*^	1.471
**AO**	100.821	4.938	89.050	2.204	101.534	3.732	85.311^*^	6.266

*Notes*: n = 3 tissues per treatment condition and number of exposures;

* = p‐value <0.05

**TABLE 5:** Percent viability by treatment condition and number of exposures vs. vehicle control (VC)

Treatment Condition	24 exposures over 4hrs		60 exposures over 10 hours		120 exposures over 20 hours		240 exposures over 40 hours	
	Mean %	SD	Mean %	SD	Mean %	SD	Mean %	SD
**IC**	100.391	2.822	103.858	3.545	90.894	1.800	115.696	2.476
**NC**	99.046	1.635	109.622	6.343	94.133	3.434	129.007	5.245
**VC**	100.000	3.191	100.000	2.087	100.000	5.610	100.000	1.898
**AO**	99.859	4.891	97.618	2.416	95.577	3.513	110.057	8.083

*Notes*: n = 3 tissues per treatment condition and number of exposures;

* = p‐value <0.05

The study had >80% power to detect a 0.2 difference in optical density between the AO‐treated cells and IC (assumes >5000 cells per well, 2‐sided 2‐sample z‐test).

## 4 DISCUSSION

The recent COVID‐19 pandemic highlights the need for safe, effective, and readily available healthcare and consumer hand antiseptics. Alcohol‐based hand rubs (ABHR), the most widely used hand hygiene products, have been scrutinized due to the lack of efficacy and safety data.^1^, ^15^ ABHR's cause skin irritation, and irritated skin can result in systemic absorption; ABHRs have other toxicities and may promote antimicrobial resistance.^16^ AO holds promise as an alternative to ABHRs.^1^

This study demonstrated the viability of human skin exposed to AO using conditions similar to how healthcare workers may perform hand hygiene. After 24, 60, and 120 7‐second exposures to AO, tissue sample viability was no different than with controls, and a 15% reduction in viability compared to untreated (negative) control by the conclusion of the test period. After 240 exposures, or a cumulative 28 minutes of exposure over four days, tissues exposed to AO showed no difference in viability compared to incubated control tissues and samples treated with dH_2_0 (VC).

Our results are consistent with a prior in vitro study evaluating human keratinocytes.^10^ In this study, a similar concentration of AO was used (4ppm) with up to 15 minutes of constant exposure. The cell survival rate was 92.4%, with no damage to or below the stratum corneum.

Further, our results are consistent with in vivo reports of repeated exposure to AO, where cellular toxicity may be reported as redness, irritation, or thickening of the skin. A systematic review of clinical studies on short‐term exposure to AO (typically 60 seconds or less) did not find reports of significant adverse effects.^17^ Interestingly, in a study comparing AO to alcohol hand disinfectant among 30 nursing students, skin irritation was reported by 20% of subjects following alcohol exposure; however, no irritation was reported from AO.^1^ Finally, we recently reported a cross‐sectional look‐back study of 30 people routinely using AO over three years for hand hygiene without a single reported adverse event.^18^

Our results should be interpreted considering several limitations. This exploratory study used a small number of tissue samples and only one concentration of ozonated water. Exposure was done over the first six minutes of the hour to limit time out of incubation. While cumulative exposure approximates the goal of real‐world hand hygiene conditions, it is more likely that hand hygiene takes place at inconsistent time intervals across a period of work. At the 40‐hour test, NCs showed higher viability than the IC, VC and AO‐exposed tissues. We speculate that this is related to mild differentiation as a result of multiple exposures to air over the course of the study.^19^ While optical density is the gold standard to measure viability in this study, new and evolving quantitative methods may be more accurate.^20^ Finally, the potential influence of penicillin/streptomycin added to the 40‐hour exposure groups on the findings should be considered. However, cell culture testing supports this approach, and we are unaware of any potential for interaction with the assay.

In summary, this well‐controlled study of full‐thickness recombinant human skin cells repeatedly exposed to AO at 4.0 ppm demonstrated high viability over a 40‐hour test period. This information helps to inform the evaluation of AO as a potential alternative for hand and wound antisepsis.

## AUTHOR CONTRIBUTIONS

RML contributed to the study conception and design. RAH, KNB, CAH, SAL, HDB, and BRL contributed to the writing and preparation of the manuscript. All authors read, edited, and approved the final manuscript.

## CONFLICT OF INTEREST STATEMENT

BRL serves on the medical advisory board of 3Oe Scientific, Inc. RML is employed by 3Oe Scientific, Inc. and holds equity in 3Oe Scientific, Inc. BRL does not hold equity.

## Data Availability

The data that support the findings of this study are available from the corresponding author upon reasonable request.
